# Adhesion Protein VSIG1 Is Required for the Proper Differentiation of Glandular Gastric Epithelia

**DOI:** 10.1371/journal.pone.0025908

**Published:** 2011-10-04

**Authors:** Odgerel Oidovsambuu, Gunsmaa Nyamsuren, Shuai Liu, Wolfgang Göring, Wolfgang Engel, Ibrahim M. Adham

**Affiliations:** Institute of Human Genetics, University of Göttingen, Göttingen, Germany; Ludwig-Maximilians University, Germany

## Abstract

VSIG1, a cell adhesion protein of the immunoglobulin superfamily, is preferentially expressed in stomach, testis, and certain gastric, esophageal and ovarian cancers. Here, we describe the expression patterns of three alternatively spliced isoforms of mouse *Vsig1* during pre- and postnatal development of stomach and potential function of *Vsig1* in differentiation of gastric epithelia. We show that isoforms *Vsig1A* and *Vsig1B*, which differ in the 3′untranslated region, are expressed in the early stages of stomach development. Immunohistochemical analysis revealed that VSIG1 is restricted to the adherens junction of the glandular epithelium. The shorter transcript *Vsig1C* is restricted to the testis, encodes an N-terminal truncated protein and is presumably regulated by an internal promoter, which is located upstream of exon 1b. To determine whether the 5′ flanking region of exon 1a specifically targets the expression of *Vsig1* to stomach epithelia, we generated and analyzed transgenic mice. The 4.8-kb fragment located upstream of exon 1a was sufficient to direct the expression of the reporter gene to the glandular epithelia of transgenic stomach. To determine the role of VSIG1 during the development of stomach epithelia, an X-linked *Vsig1* was inactivated in embryonic stem cells (ESCs). Although *Vsig1^−/Y^* ESCs were only able to generate low coat color chimeric mice, no male chimeras transmitted the targeted allele to their progeny suggesting that the high contribution of *Vsig1^−/Y^* cells leads to the lethality of chimeric embryos. Analysis of chimeric stomachs revealed the differentiation of VSIG1-null cells into squamous epithelia inside the glandular region. These results suggest that VSIG1 is required for the establishment of glandular versus squamous epithelia in the stomach.

## Introduction

The gastrointestinal tract is developed from the primitive gut tube, which comprises the endodermal epithelium and surrounding mesoderm. During embryonic development, endoderm of the foregut gives rise to the epithelia of the esophagus, stomach and duodenum, while that of the mid- and hindgut differentiate into the epithelial layer of the intestine, caecum and colon [1], [2]. In mice, the pseudostratified epithelia of the foregut transdifferentiates at embryonic day E13.5 into stratified squamous epithelia in the esophagus and forestomach, and into columnar glandular epithelia in the distal stomach [3]. The squamous epithelium of murine embryos is not keratinized and composed of multilayered epithelia, becoming keratinized at approximately 4 weeks after birth [4]. The terminal differentiation of glandular epithelia begins on E14.5 and continues into early postnatal development, where the monolayered epithelium invaginates into the neighboring mesoderm and forms a primitive gastric unites [3]. Subsequent cytodifferentiation leads to the development of different cell lineages in the gastric glands [5].

The primary structure of cell adhesion molecules that belong to the immunoglobulin superfamily (IgSF) is characterized by the presence of one or more Ig-like domains in the extracellular region that is implicated in cell-cell adhesion, a transmembrane domain, and one cytoplasmic C-terminal region. The cytoplasmic tail of adhesion molecules is linked to the actin cytoskeleton through many peripheral membrane proteins, including members of the catenin, partitioning-defective (PAR) and zonula occludens (ZO) families, which strengthen cell-cell adhesion and establish epithelial cell polarization [6]. *VSIG1/A34*, a novel member of IgSF, was first identified and characterized in humans. *VSIG1*, an X-linked gene, is predominantly expressed in stomach and testis, and is characterized by the presence of two Ig-like domains [7]. VSIG1 is related in polypeptide sequence to CTX proteins. Members of the CTX family are localized to adherens junctions between epithelial and endothelial cells of different tissues. Some CTX-like proteins are expressed in the testis, namely CAR, BT-IgSF and JAM C, while A33 is exclusively expressed in the epithelial cells of the gastrointestinal tract [8]–[12].

In the present study, we determined the expression patterns of multiple spliced transcripts of murine *Vsig1* during both prenatal and postnatal development of the stomach. To determine the 5′ region that specifically targets the expression of mouse *Vsig1* to the stomach, we generated and analyzed transgenic mice expressing enhanced green fluorescent protein (EGFP) under control of the 4.8-kb genomic sequence upstream of exon 1a. To determine the potential role of *Vsig1*, we inactivated this gene in embryonic stem cells (ESCs) through homologous recombination, generated chimeric male mice from two *Vsig1^−/Y^* ESC lines, and studied the differential patterns of *Vsig1^−/Y^* cells in gastric epithelia.

## Results

### Identification and characterization of *Visg1*

**Figure 1:**
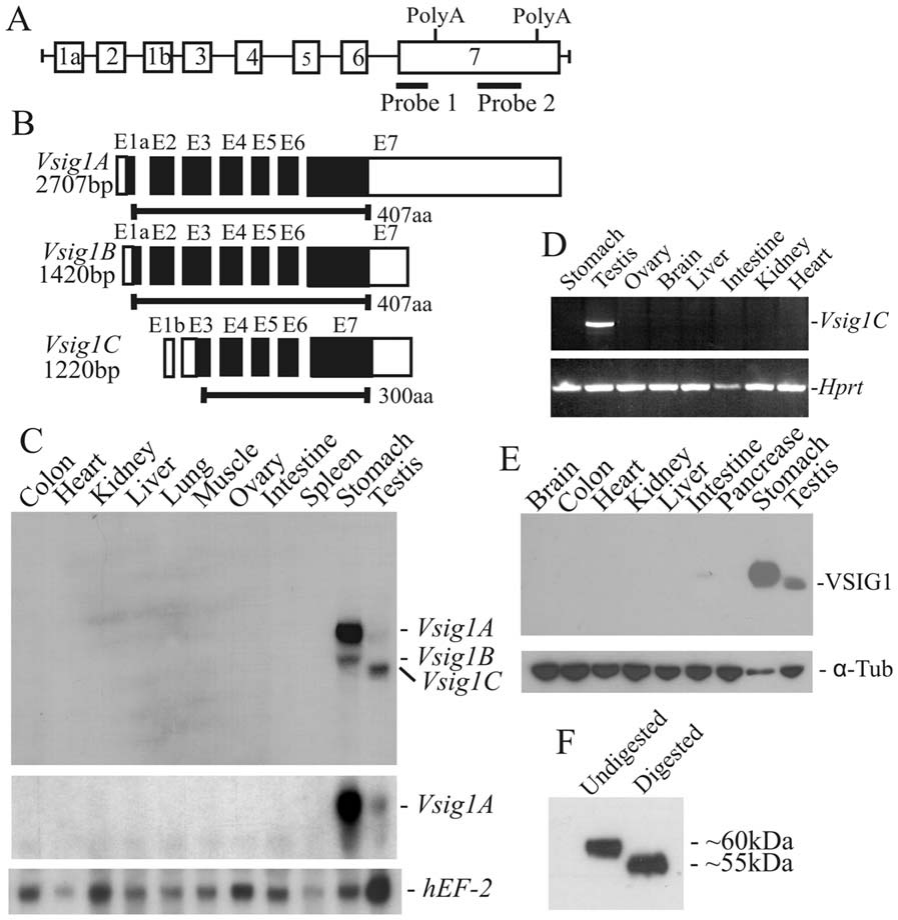
Characterization and expression analysis of *Vsig1* splice variants. (A) Schematic diagram of the *Vsig1* gene. Boxes and lines represent the exons and introns, respectively. Positions of both polyA signals and probes used in Northern blot analysis are shown. (B) Schematic representation of exonic sequences present in the different *Vsig1* mRNA isoforms. Black boxes represent the coding exon, while white boxes represent the 5′ and 3′UTRs of the *Vsig1* splice variants. (C) Northern blot with total RNA from different tissues of 3-month-old mice was hybridized with probe 1 (top panel) and probe 2 (middle panel). Integrity and variation of loaded RNA samples were assessed by rehybridization with a probe for human elongation factor 2 (EF-2). (D) Restricted expression of *Vsig1C* isoform in testis was confirmed by RT-PCR analysis using primers containing the sequence of exons 1b and 4. The used primers only amplify the 396-bp cDNA fragment in testis RNA. Production of the control *Hprt* products was observed throughout tissues, demonstrating the presence of intact loaded RNA. (E) Immunoblot with cellular extracts from different tissues was probed using polyclonal anti-VSIG1 antibodies and subsequently reprobed with monoclonal anti- α-tubulin antibodies (α-Tub). (F) Immunoblot with untreated and N-glycosidase F-treated stomach extracts was probed with anti-VSIG1 antibodies.

We used subtractive cDNA hybridization to isolate cDNA clones that are exclusively expressed in the stomach. Stomach cDNAs were hybridized with abundant intestine and liver cDNAs, and subtractive cDNA fragments were cloned and sequenced. A 300-bp cDNA fragment of one clone (Sx) hybridized by Northern blot analysis with a 2.7-kb transcript in stomach RNA. A NCBI database search revealed that the identified cDNA was identical to a sequence in the 3′ untranslated sequence of *Vsig1* gene (accession No. NP_084457). Alignment of different cDNA sequences in this database suggested that *Vsig1* is transcribed into three splice variants, which we designate as *Vsig1A* (2.18-kb; AK019565.1), *B* (1.42-kb; AK160478.1) and *C* (1.22-kb; AK160478.1). The cDNA sequence of *Vsig1A* contains two predicted polyadenylation sites that span 0.6-kb and differs from that of *Vsig1B* in the length of the 3′ untranslated region (UTR; Fig. 1A and B). The *Vsig1C* isoform contains a 61-bp sequence at the 5′UTR, which is transcribed from alternative exon 1b, located in intron 2 of the *Vsig1* gene (Fig. 1A and B). The *Vsig1A* and *B* variants are transcribed by exons 1a and 2 through 7, whereas *Vsig1C* contains the sequences of exons 1b and 3 through 7 (Fig. 1A). The coding sequences of the three splice isoforms are located in the same reading frame, but VSIG1A and VSIG1B have an additional 107 amino acids at their N-termini. The predicted amino acid sequence of VSIG1C lacks the signal peptide sequence and the first Ig-like domain, suggesting that this truncated protein is present in the cytoplasm.

To determine the tissue distribution of the *Vsig1* transcripts, Northern blot and RT-PCR analyses were performed with total RNA from different tissues of 3-month-old mice. The cDNA probe (probe 1 in Fig. 1A) only recognized the 2.7-kb *Vsig1A* and 1.7-kb *Vsig1B* transcripts in stomach, and the 2.7-kb *Vsig1A* and 1.4-kb *Vsig1C* transcripts in testis (Fig. 1C, top panel). No *Vsig1* mRNAs could be detected in other tissues. Expression levels of *Vsig1A* in the stomach were relatively higher than that of *Vsig1B*, while expression levels of *Vsig1C* were markedly higher than *Vsig1A* in the testis. To confirm that *Vsig1B* and *C* mRNA isoforms result from alternative splicing of the 3′UTR, an RNA blot was hybridized with cDNA probe 2 (Fig. 1A). Only the *Vsig1A* transcript could be detected in stomach and testis (Fig. 1C, middle panel). To validate the presence of exon 1b in *Vsig1C* mRNA, RT-PCR was performed with RNA from different tissues. Primers containing the sequences of exons 1b and 4 could only amplify a fragment in testicular RNA, confirming the presence of exon 1b in the testis-specific *Vsig1C* transcript (Fig. 1D).

We raised rabbit polyclonal antibodies against the intracellular domain of VSIG1. The affinity-purified antibody only recognized a 60-kDa protein in stomach and a 55-kDa protein in testis. No additional proteins of smaller size, which may be the products of the *Vsig1C* transcript, could be detected in extracts of adult testis (Fig. 1E). The sequence analysis predicted that the deduced proteins of the *Vsig1A* and *B* variants have a molecular mass of 47-kDa and an extracellular domain that bears three NX(S/T) motifs for *N*-linked glycosylation. Therefore, we enzymatically digested the stomach lysates with *N*-glycosidase F to release putative *N*-linked oligosaccharides. After treatment, the molecular mass of VSIG1 shifted from 60- to 55-kDa, confirming that the 60-kDa protein recognized by the antibody in the stomach is N-glycosylated VSIG1 (Fig. 1F).

### Expression pattern of Vsigl during stomach development

**Figure 2:**
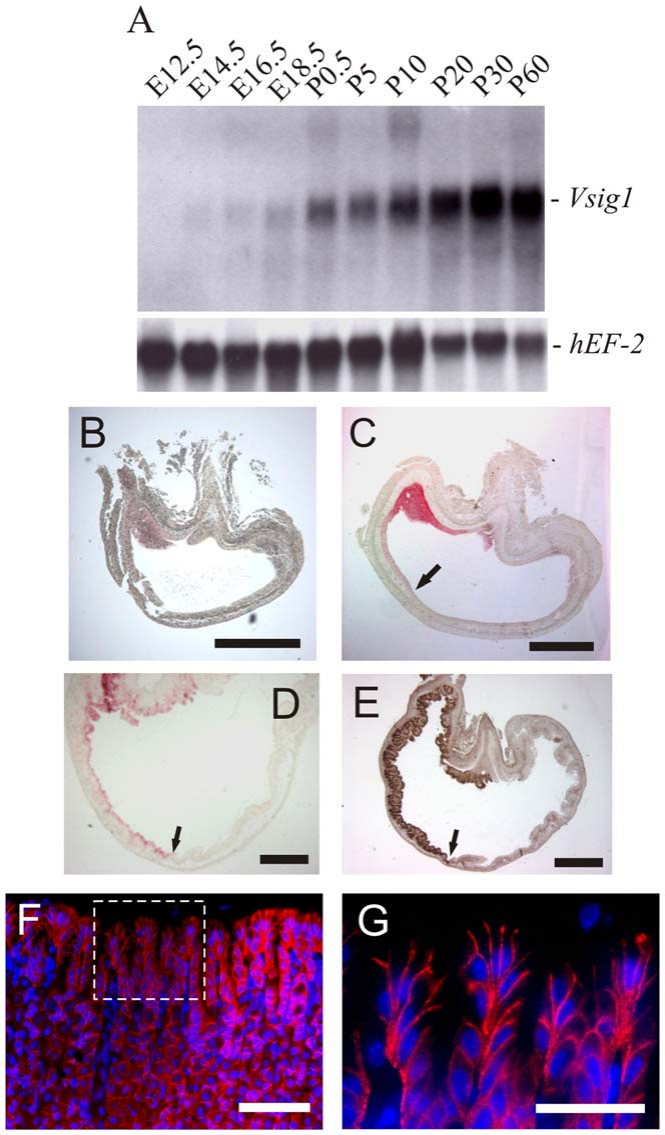
Expression analysis of VSIG1 during stomach development. (A) RNA blot of total RNA isolated from the stomach of different stages of pre- (E) and postnatal (P) development was hybridized with *Vsig1* (probe 2 in Fig. 1A) and the *hEF-2* cDNA probe. (B) Immunohistochemistry of paraffin sections with anti-VSIG1 antibody shows the restricted expression of VSIG1 in the glandular epithelium of the stomach at E12.5 (B), E13.5 (C) and E17.5 (D). (E) Expression of GATA4 in glandular epithelia of the stomach at E17.5. Arrows in C–E mark the transitional junction between the glandular and squamous epithelia. In 3-month-old stomachs, VSIG1 is located at the adhesion junctions between epithelial cells of the gastric unit (F). The box in F is magnified in G and shows restricted localization of VSIG1 to the basolateral membrane of pit cells (G). Scale bar (B–E) = 500 µm; (F) = 100 µm; (G) = 20 µm.

Given the high levels of *Vsig1* mRNA detected in the stomach, we performed analyses to determine both the developmental expression and cellular distribution of VSIG1 in this tissue. Northern blot analysis showed high expression levels of *Vsig1* in the stomach at E14.5, and the expression levels were maintained throughout the neonatal and adulthood stages (Fig. 2A).

**Figure 3:**
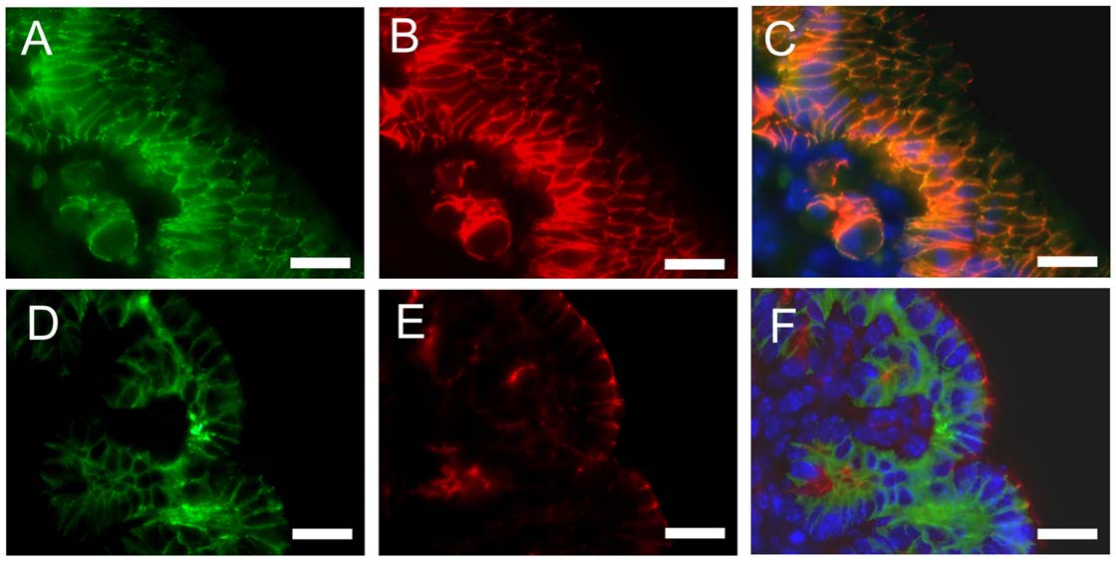
Subcellular localization of VSIG1 to adherens junctions of glandular epithelia. Double immunostaining of embryonic stomach (E17.5) with rabbit polyclonal anti-VSIG1 (A and D), mouse monoclonal anti-α-catenin (B) and mouse monoclonal anti-ZO1 (E) antibodies. The immunoreactivity for VSIG1 is distributed across the basolateral membranes of luminal epithelia and cell-cell adhesion sites, and overlap with that of α-catenin (C). Immunostaining signals of ZO1 are restricted to tight junctions of luminal epithelia (E) and do not show co-localization with VSIG1 (F). Nuclei (C and F) are visualized with DAPI (blue). Scale bar = 20 µm.

The murine stomach develops from homogeneous pseudoepithelium that differentiates into squamous epithelium in the forestomach region and into glandular epithelium in the corpus and antrum regions of the stomach. The cellular distribution of VSIG1 in the embryonic stomach was determined by immunohistology. Only negligible staining could be observed in the epithelium of the stomach at E12.5 (Fig. 2B). At E13.5, VSIG1 expression was detected in the glandular epithelium of the corpus and antrum regions of the stomach but not in the squamous epithelium of the forestomach (Fig. 2C). In the stomach at E17.5, much higher expression levels of VSIGl were observed in the primordial buds of the glandular gastric epithelium (Fig. 2D). Staining of stomach at E17.5 with anti-GATA4, a marker of glandular epithelia, confirmed the restricted expression of VSIGl in the glandular epithelium (Fig. 2E). We examined the expression patterns of VSIGl in the gastric gland of adults using immunohistochemistry. The VSIGl protein was localized in the plasma membranes of all cells of the gastric gland (Fig. 2F) and was restricted to the basolateral membranes of pit cells (Fig. 2G). No VSIG1-immunostaining could be detected in the squamous epithelium of the forestomach (data not shown). To determine the subcellular localization of VSIG1, we performed co-immunolocalization of VSIG1 with β–catenin and ZO-1, which are known to be localized in the adherens junction and tight junction, respectively. VSIG1 was localized in the basolateral membranes of luminal epithelia similar to that of the adherens junction-associated β-catenin protein (Fig. 3A–C). In contrast, ZO-1 levels were higher in the apex of the lateral membrane (Fig. 3D–F). Overlay images did not show co-localization of VSIG1 immunostaining with ZO-1. These results reveal that VSIG1 is present in adherens junctions of glandular epithelia.

### Expression of Vsig1-EGFP transgenic allele during stomach development

**Figure 4:**
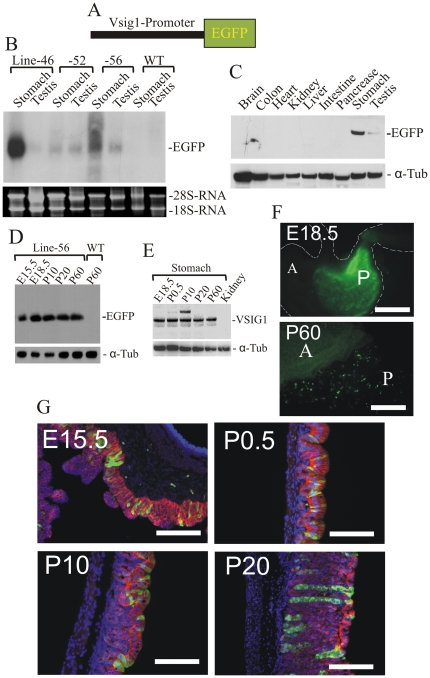
Generation and expression analysis of the *Vsig1-EGFP* transgenic allele. (A) Schematic representation of the *Vsig1-EGFP* transgenic construct. The *Vsig1-EGFP* construct consists of the 4.5-kb genomic fragment located upstream of exon 1a of the *Vsig1* gene (black box) and the EGFP gene (green box). (B) Expression of the *Vsig1-EGFP* transgenic allele in adult stomachs and testes of different transgenic lines and wild-type (WT) mice was determined by Northern-blot hybridization using the *EGFP* probe. Integrity of RNA samples was documented by images of the corresponding agarose gel. (C) Immunoblot of EGFP expression in cellular extracts from different tissues of 3-month-old transgenic mouse. The protein blot was subsequently probed with anti-α-tubulin antibody. (D) Expression of the *Vsig1-EGFP* transgenic allele during pre- and postnatal stomach development was examined by immunoblotting using total lysates obtained from transgenic stomachs of embryos at E15.5 and E18.5, and from P10, P20 and P60 mice. Protein extract from wild-type stomach (WT) was used as controls. (E) Temporal expression of VSIG1 during prenatal and postnatal development of stomach was examined by immunoblotting. (F) Fluorescent micrographs of stomachs from transgenic embryos at E18.5 and 60-day-old mice show EGFP epifluorescence in the posterior stomach (P) but not in the anterior stomach (A). (G) Expression of Vsig1-EGFP in the glandular epithelium was confirmed by immunofluorescence in paraffin sections of E15.5, P0.5, P10 and P20 with anti-EGFP (green fluorescence) and anti-VSIG1 (red fluorescence) antibodies. DAPI (blue fluorescence) was used for nuclear staining. Scale bar (F) = 500 µm; (G) = 200 µm.

Sequence and expression analyses suggest that the promoter regulating the expression of *Vsig1A* transcripts in gastric epithelia is located in the 5′ flanking region of exon 1a. To determine whether the 5′ flanking region of exon 1a can regulate the expression of EGFP exclusively in glandular epithelia, a *Vsig1-EGFP* reporter was constructed using a 4.8-kb genomic fragment located upstream of the mouse *Vsig1* translation start codon (Fig. 4A). Injection of the *Vsig1-EGFP* reporter construct into fertilized oocytes was performed to generate transgenic mice. Three transgenic founders showed germ-line transmission of the *Vsig1-EGFP* transgenic allele. Northern and Western blot analyses revealed the high expression of EGFP mRNA and protein in the stomachs and testes of transgenic mice (Fig. 4B and C). The temporal regulation of EGFP and VSIG1 was analyzed by Western blot using stomach extracts from sequential time points during fetal and postnatal development. In contrast to dramatically increase in mRNA expression of *Vsig1* during stomach development (Fig. 2A), relatively equal levels of EGFP and VSIG1 protein were expressed in stomachs of mice at both fetal and postnatal stages (Fig. 4D and E). These results suggest that the expression of *Vsig1* is under post-transcriptional control. Expression of EGFP *in vivo* was initially investigated in freshly collected tissues from embryos at E18.5 and adult mice (Fig. 4F). EGFP epifluorescence was observed in embryonic and adult stomachs, while no other embryonic or adult tissues, including testis, exhibited any GFP fluorescence above background (data not shown). EGFP fluorescence was uniformly distributed in the distal region of the embryonic stomach, whereas EGFP signals were restricted to discrete spots along the corpus region of adult stomachs (Fig. 4F). This expression pattern was observed in all three transgenic lines. To determine whether the expression of EGFP in embryonic and postnatal stomachs resembles that of endogenous VSIG1, immunohistological analysis was performed using anti-EGFP and -VSIG1 antibodies (Fig. 4G). In contrast to high levels of EGFP expression in transgenic stomachs shown by western blot analysis, the used anti-EGFP antibody could only detect a few EGFP-positive cells, which were distributed along the glandular epithelia (Fig. 4G).

### Targeted disruption of *Vsig1*

**Figure 5:**
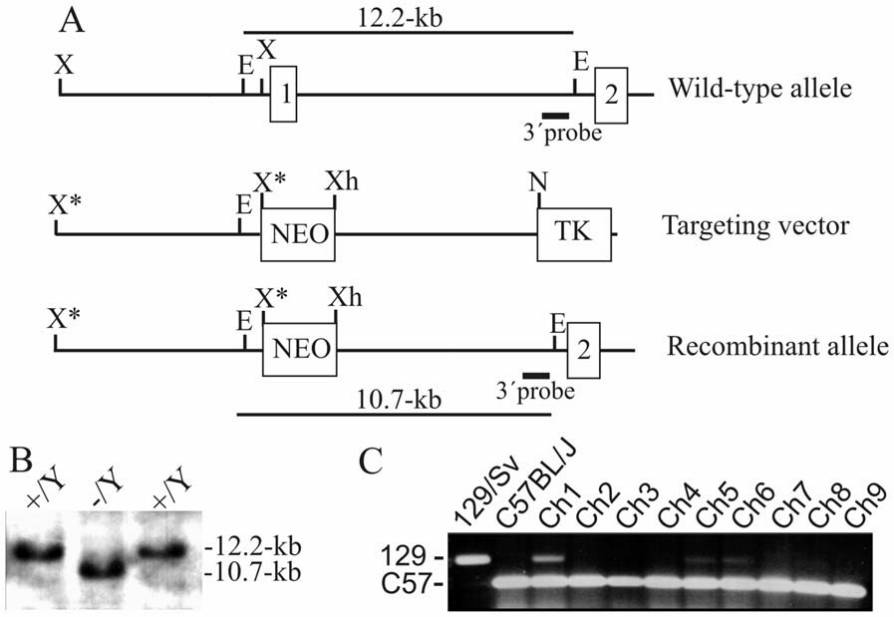
Targeting disruption of the *Vsig1*. (A) Structure of the wild-type, targeting vector and recombinant allele are shown together with the relevant restriction sites. A 2.5-kb genomic fragment containing exon 1a was replaced by a *pgk-neo* selection cassette (NEO). The probe used and predicted length of the *Eco*RI restriction fragment in Southern blot analysis are shown. TK, thymidine kinase cassette; E, *Eco*RI; X, *Xba*I; X*, disrupted *Xba*I site; Xh, *Xho*I. (B) Blot with *Eco*RI-digested genomic DNA of recombinant ESC clones was probed with the 3′ probe shown in panel A. The external probe recognized only a 10.7-kb fragment of recombinant allele in *Vsig1^−/Y^* ESCs and a 12.2-kb fragment of the wild-type allele in *Vsig1^+/Y^* ESCs. (C) PCR assay using microsatellite markers was performed to determine the degree of chimerism in the stomachs of chimeric male mice. The 129- and C57-specific fragments were amplified using DNA of the 129/Sv and C57BL/6J mouse strains, and stomach isolated from different chimeric males (Ch).

To clarify the *in vivo* function of *Vsig1*, we disrupted the X-linked *Vsig1* gene in the XY ESCs using a replacement targeting strategy (Fig. 5A). A targeting construct was designed to replace a 2.5-kb genomic fragment containing exon 1a with a neomycin resistance gene (*neo*). Exon 1a contains the translation initiation codon, ATG, and the expected targeting event would generate an allele for an untranslated *Vsig1* mRNA. Following electroporation and drug selection, homologous recombinants were selected by Southern blot analysis of *EcoRI*-restricted genomic DNA, using a 5′ external probe (Fig. 5B). The external probe detected only a 10.7-kb recombinant fragment in the *Vsig1^−/Y^* ESCs and a 12.2-kb wild-type fragment in non-homologous recombinants. Injection of two *Vsig1^−/Y^* ESC clones in blastocysts resulted in 38 male and 12 female chimera, in which the chimerism ranged from 5–35% according to coat color. No male chimeras transmitted the targeted allele to their offspring, and the average litter size was not significantly different from that obtained from breeding 129/Sv males with C57BL/6J females. In contrast, chimeric males, which were generated by injection of blastocysts with non-homologous recombinant ES cell line, did give germ line transmission to their offspring. Karyotyping of both Vsig1-null ES cell lines showed no chromosomal abnormalities (data not shown). These results suggest that the failure to generate chimeric males with high chimerism was not due to ES defects or technical procedure, but to the death of chimeric embryos with a high contribution of *Vsig1^−/Y^* cells. To address this question, we have sacrificed 3 pregnant females at gestational day 17.5. Seven live embryos were found, and no signs for reabsorbed embryos could be detected in uteri of foster females. Genotyping of these embryos revealed a low contribution of *Vsig1^−/Y^* cells in two chimeric embryos.

### Transdifferentiation of *Vsig1^−/Y^* cells into squamous epithelia in the corpus region of the stomach

**Figure 6:**
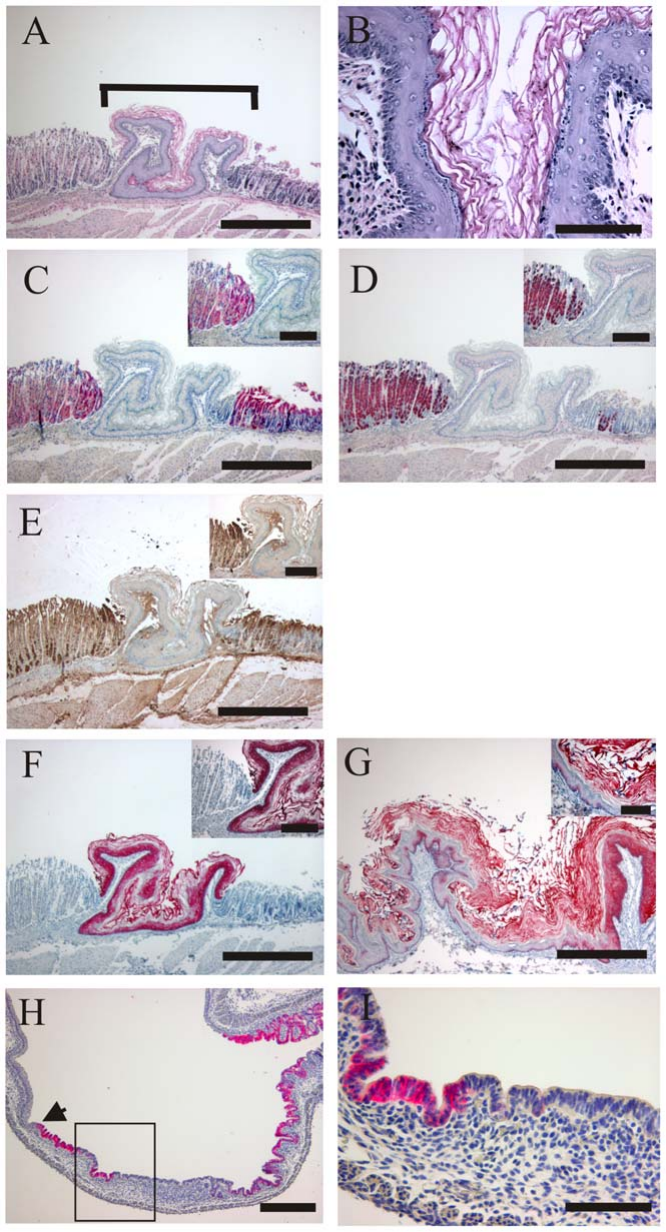
Transdifferentiation of the *Vsig1^−/Y^* cells into squamous epithelia inside the gastric corpus of chimeric *Vsig1^−/Y^↔Vsig1^+/Y^* stomachs. (A–F) Serial sections of stomach prepared from 5-month-old chimeric mice were stained with H&E (A and B) or immunohistologically analyzed for expression of glandular and squamous epithelia-specific markers (C–F). The bracket in H&E-stained sections (A) marks the area containing squamous epithelia, which are present inside the glandular epithelia of the gastric corpus and is magnified in B. Cells of this lesion do not express VSIG1 (C) or glandular epithelium-specific markers H^+^,K^+^-ATPase (D) and GATA4 (E), but do express cytokeratin K5 (F), which is normally expressed in squamous epithelia of the forestomach (G). Inserts show higher magnification. (H and I) Dissected stomachs from *Vsig1^−/Y^↔Vsig1^+/Y^* chimeras at E17.5 were longitudinally sectioned and immunological stained with anti-VSIG1 antibody. The transition zone between glandular mucosa and stratified squamous epithelia of the forestomach is indicated by an arrow. The box in H is magnified in I and denotes a patch of atypical squamous epithelium that lacks the primordial gastric units of the glandular epithelium as well as cells that express VSIG1. Scale bar (A and C–H) = 500 µm; (B and I) = 100 µm; inserts = 200 µm.

To evaluate the consequences of *Vsig1* deficiency on stomach development of chimeric mice, we first performed a PCR assay based on microsatellite polymorphisms to determine the contribution of the 129/Sv and C57BL/6J cells in testes and stomachs isolated from 18 chimeras. As shown in Figure 5C, the ratio of *Vsig1^−^* ^/Y^ cells as judged by the level of the amplified 129/Sv-specific fragment was relatively higher in stomachs of two chimeras, suggesting the contribution of *Vsig1^−^* ^/Y^ cells in stomach development of both chimeras. Next, we analyzed the expression of VSIG1 protein in the *Vsig1^−^* ^/Y^↔*Vsig1^+^* ^/Y^ chimeric stomachs. We found that some areas of glandular epithelia in the corpus region contain VSIG1-negative cells (Fig. 6C). In contrast to the neighboring *Vsig1^+^* ^/Y^ epithelium, the VSIG1-deficient epithelium did not express either the H^+^/K^+^ -ATPase P-subunit and GATA4, which are markers of parietal cells in the glandular epithelium (Fig. 6D and E). Hematoxylin and eosin (H&E) staining revealed that VSIG1-negative epithelia in the corpus region of the stomach was morphologically differed from adjacent *Vsig1^+/Y^* epithelia and had an atypical morphology of the squamous epithelium, which is normally localized in the forestomach (Fig. 6A and B). Immunohistochemical analysis revealed that cytokeratin-5 is highly expressed in the heterotypic epithelium as well as in the squamous epithelium of the forestomach (Fig. 6F and G). To further confirm that VSIG1 is required for proper the differentiation of glandular epithelia, we performed immunohistological analysis on stomach sections of E17.5 chimeric embryos. Primordial buds of gastric units were formed in the glandular epithelium of wild-type embryos and in VSIG1-positive epithelia of chimera stomachs (Fig. 6H). In contrast, epithelium in the lesion containing VSIG1-null cells did not display primordial gastric glands development (Fig. 6H and I). These results further demonstrate that VSIG1 is required for the establishment of glandular identity.

## Discussion

In this report, we investigated the expression patterns of mouse *Vsig1* during stomach development, and determined the consequences of *Vsig1*-deficiency on the differentiation of stomach epithelia. We found that the alternative usage of two promoters and their own first exons leads to the transcription of stomach- and testis-specific *Vsig1* mRNAs. The *Vsig1A* and *B* transcripts, which differ in the lengths of their 3′UTRs, are regulated by sequences located in the 5′flanking regions of exon 1a. Results of expression studies revealed that *Vsig1A* is specifically expressed in stomach epithelia. The shorter *Vsig1C* transcript is exclusively expressed in testis and is presumably directed by sequences located in the 5′flanking region of internal exon 1b (our unpublished data).

VSIG1 is broadly distributed along the lateral membrane of glandular epithelia in a distribution similar to that of the adherens junction-associated protein β-catenin. In contrast, VSIG1 distribution in the luminal epithelium did not co-localize with the tight junction protein ZO1. Recent report revealed the interaction of VSIG1 with ZO-1 by co-immunoprecipitation analysis [13]. Similar patterns of subcellular localization were reported for ESAM, which is another member of the CTX family [14].

*Vsig1* expression begins in the early stages of stomach development, is restricted to glandular epithelia of the corpus and antrum regions, and is absent from the squamous epithelium of forestomach. Around E12.5, the epithelium that lines the mesenchyme of the developed stomach and is composed of homogeneous pseudostratified epithelia begins to cytodifferentiate along the proximal-distal axis into squamous multilayered epithelia in the forestomach and columnar epithelia in the glandular stomach. The differentiation of VSIG1-null cells into squamous epithelia in the corpus region of the chimeric stomach revealed that VSIG1 is required for the proper differentiation of glandular epithelia of the stomach. Expression of the squamous epithelial marker cytokeratin 5 in *Vsig1*-deficient lesions indicated that the differentiation program of squamous epithelium is ectopically activated in the glandular region of the stomach. These results suggest that VSIG1 is included in a signal pathway that represses a differentiation program of the squamous epithelium in the distal stomach. Several signaling molecules and transcription factors are essential for controlling the epithelial differentiation of the stomach. Mutations in several genes result in alternations of the sharp boundary between the multilayered squamous epithelia and the glandular monolayer. Mice with a tissue-specific loss of the nuclear hormone receptor *Coup-TFII*, with a hypomorphic allele of *Sox2* and a mutation in *Shh* showed expansion of glandular epithelia in the region of the forestomach [15]–[17]. In contrast, embryos with mutations in activin receptor type II (*ActR*), *FGF10* and its receptor *FGFR2b* develop multilayered squamous epithelia in the corpus of the distal stomach [18], [19]. The differentiation of *Vsig1^−/Y^* cells into squamous epithelia inside the glandular region of the chimeric stomach resembles that of the chimeric *Gata4^+/+^↔Gata4^−/−^* stomach [20]. The development of squamous epithelia at ectopic sites in the gastrointestinal tract is also shown in *Cdx2^−/−^↔Cdx2^+/+^* chimeric mice [21]. CDX2-null cells have been shown to form areas of ectopic forestomach in the terminal ileum of the paracaecal region and in the caecum of chimeric mice [21]. The conditional ablation of CDX2 from early stages of endoderm development results in the transdifferentiation of intestinal epithelia into squamous epithelia. These results confirm that the transcription factor CDX2 represses the differentiation program of squamous epithelia in the intestine [22]. Although there are several mouse models that show the transdifferentiation of monolayers into multilayered epithelia, there is little understanding of the molecular genetic changes that initiate and promote such epithelial transdifferentiation.

Despite several attempts to generate chimeric mice by injection of *Vsig1^−/Y^* cells in blastocysts, we have been able to establish chimeric mice with low chimerismus. In addition, genotyping analysis did not detect either neonatal or late embryonic death of chimeras with a high contribution of Vsig1-null cells. These results suggest that VSIG1 is necessary for early embryonic development, However, RNA and protein analysis failed to detect the expression of Vsig1 during post-implantation stages (E7.5–E12.5) and in embryonic stem cells (data not shown). Insertion of *pgk-neo* selection cassette in the 5′region of *Vsig1* mutant allele may interfere with the expression of neighboring genes. Miss-expression of neighboring genes might be the reason for limited contribution of *Vsig1^−/Y^* cells in chimeric mice. Therefore, the production of conditional knockout mice may be a promising method for direct assessment of *Vsig1* function during embryogenesis, and during testis and stomach development.

Analysis of *Vsig1-EGFP* transgenic lines revealed that the 4.8-kb sequence located upstream of exon 1a was able to direct the specific expression of the reporter EGFP in glandular epithelia of the stomach and in testis. Temporal expression of EGFP protein during stomach development was similar to that of VSIG1. Furthermore, Immunohistochemical analysis showed that EGFP is distributed along the glandular epithelium. Thus, the 5′ flanking region of *Vsig1*, which we have characterized here, will provide a useful tool to direct the specific expression of genes during embryonic and postnatal development of gastric epithelia *in vivo*.

## Materials and Methods

This study was carried out in strict accordance with the recommendations in the Guide for the Care and Use of Laboratory Animals of the National Institutes of Health. The protocol was approved by the Committee on the Ethics of Animal Experiments of the University of Göttingen (Permit Number: 33.42502/01-53.05).

### Cloning of *Vsig1* cDNA

A subtractive hybridization cDNA library was constructed using the PCR-Select cDNA subtraction kit (Clontech Laboratories Inc., Palo Alto, CA) according to the manufacture's instructions. Total RNA from the corpus region of the stomach, liver and intestine was isolated and reverse transcribed. Stomach cDNA was used as the tester, while intestine and liver cDNA served as the drivers. Tester and driver cDNAs were hybridized, and the remaining unhybridized sequences were amplified by PCR, cloned into the pGEM-T easy vector (Promega Corp) and sequenced.

### RNA analysis

Total RNA was extracted from different tissues using a Qiagen RNA kit (Qiagen, Hilden, Germany). For Northern blot analysis, 15 µg RNA were electrophoresed in 1.2% agarose gels containing 2.2 M formaldehyde, transferred into nylon membranes and hybridized with the P- labeled cDNA probe. *Vsig1* cDNA fragments, which were used in expression analyses, were amplified by RT-PCR using stomach RNA as a template. A 293-bp fragment containing a part of the 3′ UTR of *Vsig1* cDNA was amplified using the MK4-F1 primer (5′-GTACTAATATGTAACATAACA-3′) and the MK4-R1 primer (5′- GTACCAATTGATACATACCTT-3′). A second 567-bp cDNA containing the sequence encoding the cytoplasmic domain of *Vsig1* was amplified using the SX-FuProF1 primer (5′-GGCGGATCCGCTGCTGTTATCATCTGTGTGG-3′) and SX-FuProR1 primer (5′-GGGCTCGAGGGGAATTGCCATACTAAATGC-3′). Both amplified cDNAs were cloned and sequenced. For expression analysis of *Vsig1* during early embryonic development, RT-PCR assays were performed using 2 µg total RNA and the One Step RT-PCR Kit (Qiagen). Primers to amplify the *Vsig1* cDNA fragment containing the sequence from exons 1b to 4 were SX-RTF1b (5′-CTGGCCAGAGGCATAGGTTG-3′) and SX-RTR4 (5′-GGCACAATGGTGTTCCCCTCA-3′).

### Antibodies

Rabbit antibodies against the cytoplasmic domain of VSIG1 were raised against the GST-VSIG1 fusion protein. To generate the fusion protein, the 189-bp cDNA fragment that encodes the cytoplasmic domain of VSIG1 was amplified using the SX-FuProF1 and SXFuProR1 primers and cloned into the pPET-41a vector (Novagen, Darmstadt, Germany). Rabbits were immunized with the GST-VSIG1 fusion protein mixed with Freund's adjuvant (Sigma-Aldrich, Munich, Germany). The antibodies were affinity purified by absorption onto the same fusion protein covalently coupled to Affi-Gel 10 agarose beads (BIO-RAD, Hercules, CA). The following commercially available antibodies were used: rabbit anti-GATA4 (N-60; Santa-Cruz Biotechnology, Santa Cruz, CA), mouse anti-cytokeratin5/6 (clone D5/16 B4; DakoCytomation, Glostrup, Denmark), mouse anti-H^+^/K^+^ATPase (Acris, Hiddenhausen, Germany), mouse anti-GFP (Chemicon, Temecula), mouse anti-β-catenin (clone 14, BD Bioscience), mouse anti-ZO1 (clone 1A/2, Invitrogen) and mouse anti-α tubulin (Sigma). Secondary antibodies were purchase from Sigma-Aldrich.

### Histological analysis

Animals were sacrificed by cervical dislocation. Stomachs were isolated, fixed in 4% paraformaldehyde for 24 h at 4°C and embedded in paraffin. Mounted sections were deparaffinized, rehydrated, and stained with H&E.

### Western blotting and immunohistochemistry

Tissues were lysed in RIPA buffer (Santa Cruz Biotechnology). Proteins were resolved on SDS/PAGE and transferred onto nitrocellulose membranes (Amersham Bioscience, Braunschweig, Germany). Blots were blocked with 5% skim milk in Tris buffered saline/Tween-20 before incubation with the primary antibodies in blocking solution overnight at 4°C. After the washing step, bound antibodies were detected using horseradish peroxidase-conjugated anti-rabbit and anti-mouse IgGs and enhanced chemiluminescence (Pierce Chemical, USA). The primary antibodies and dilutions used were rabbit anti-VSIGl at 1∶1000 and anti-α tubulin at 1∶10000.

For enzymatic digestion of the N-linked oligosaccharides, stomach lysates were treated with N-glycosidase F enzyme (Roche, Mannheim, Germany) according to the manufacturer's instructions. The samples were then subjected to western blot analysis.

For immunohistochemistry, sections were preincubated for 1 h with 10% normal goat serum in 0.05% Tween-20/phosphate buffered saline (PBS) and incubated overnight at 4°C with 1∶500- diluted primary antibodies, washed in PBS, and then incubated with alkaline phosphatase-conjugated secondary antibodies at a 1∶500 dilution for 1 h at room temperature. After washing in PBS, the immunoreactivity was detected by incubating the sections in a solution containing Fast red TR/naphthol AS-MX phosphate tablets (Sigma).

### Generation of Vsig1-EGFP transgenic mice

The 4.5-kb *Xba*I fragment containing a sequence of the 5′flanking region was isolated from the PAC clone (MPMGc121B114407Q2, RZPD) and subcloned into *Xba*I-digested pBluescript-II(SK)+ to produce clone T1. The 350-bp genomic fragment, which contains the sequence between the 4.5-kb XbaI fragment and the translation start codon, was amplified using the primers 5′- GGAAAGTGTTACTGGAAATGTCC-3′ and 5′- AAGTCGATGCCCTTCAGCTCGA-3′. The 350-bp amplified fragment with an artificial *Not*I site at 5′-end and an *Sst*I site at 3′-end was inserted into the *Not*I/*Xba*I-digested clone T1 to create clone T2. To generate the *Vsig1-EGFP* construct, a 4.8-kb genomic fragment was isolated from clone T2 by digestion with *Sal*I/*Sst*I and subcloned into the *Sal*I/*Sst*I digested pEGFP-1 vector (Clontech). The fragments containing *Vsig1-EGFP* were separated from the construct by *Pvu*I, purified by agarose gel electrophoresis, and microinjected into fertilized FVB/N eggs to generate transgenic mice. Mice were genotyped for the presence of the transgene by PCR designed to amplify the region spanning the junction between the mouse *Vsig1* promoter and the EGFP using primers 5′-GTTCAGAGAGAACTCAGTGCCC-3′ and 5′-CGCTGAACTTGTGGCCGTTTAC-3′. Thermal cycling was carried out for 35 cycles, denaturation at 94°C for 30 sec, annealing at 58°C for 30 sec, and extension at 72°C for 45 sec.

### Generation of *Vsig1* chimeric mice

The *Vsig1*-targeting vector was constructed using the plasmid vector pPNT [23]. To construct the targeting vector, a genomic 4.5-kb *Xbal* fragment containing the sequence of the 5′-flanking region of *Vsig1* was isolated from the PAC clone (MPMGc121B114407Q2, RZPD) and cloned into the pZERO-TM-2 vector (Invitrogen). The 2,9 kb *Xho*1/*Not*1 fragment containing a sequence of intron 1 was amplified by PCR using primers SX3-ArmF1 (5′-CTGGGAAGCCTTCACTTCTCTAAG-3′) and SX3-ArmR1 (5′-TCTGCxTGAACATCAATTTGTGCTA-3′), cloned into the pGEMT-easy vector and sequenced. To generate the Vsig1-targeting construct, the 4.5-kb *Xba*I fragment was cloned into the BamH1/*Eco*R1-digested pPNT-1 vector by blunt-end ligation. Finally, the 2.9-kb *Xho*1/*Not*1 fragment was cloned into the *Xho*1/*Not*1 digested pPNT-1 vector. The resulting *Vsig1*-targeting vector was linearized with *Not*I and then transfected into RI ESCs [24].

Genomic DNA extracted from individual drug-resistant clones was screened for homologous recombination by Southern blot analysis. DNA was digested with *Eco*R1, and probed with a 3′external probe. Cells from two homologous recombinant ES-clones were injected into C57BL/6J blastocysts and these were transferred into DBA/BL6 pseudopregnant females. Chimeras were bred with C57BL/6J mice to identify ES-derived agouti offspring.

#### Determination of the contribution of the 129/Sv cells to chimeras

Genomic DNA was extracted from different chimeric mouse tissues by using the DNeasy-Kit (Qiagen). We examined the contribution of the 129/Sv cells to tissues of chimeric animals by PCR using primers (5′-GGCTTCGACCCTGGTTTTAG-3′) and (5′-TGAAAGTTCAGATGACCACACG-3′), which amplify a microsatellite in the D2Mit94 locus of mouse chromosome 2 [25]. The PCR products were 194-bp for 129/Sv and l60-bp for C57BL/6J.
